# Reversal of homocysteine-induced neurotoxicity in rat hippocampal neurons by astaxanthin: evidences for mitochondrial dysfunction and signaling crosstalk

**DOI:** 10.1038/s41420-018-0114-x

**Published:** 2018-10-22

**Authors:** Xian-jun Wang, Wang Chen, Xiao-ting Fu, Jin-kui Ma, Mei-hong Wang, Ya-jun Hou, Da-chen Tian, Xiao-yan Fu, Cun-dong Fan

**Affiliations:** 1 Department of Neurology, People’s Hospital of Linyi Affiliated to Qingdao University, Linyi, 276000 Shandong China; 2 School of Basic Medicine, Taishan Medical University, Taian, Shandong 271000 China; 3 Faculty of Bioresource Sciences, Akita Prefectural University, 241-438 Kaidobata-Nishi, Shimoshinjo-Nakano, Akita-shi, Akita 010-0195 Japan; 4 Department of Neurology, People’s Hospital of Yishui, Linyi, 276400 Shandong China

**Keywords:** Cellular neuroscience, Apoptosis

## Abstract

Elevated plasma level of homocysteine (Hcy) represents an independent risk for neurological diseases, and induction of oxidative damage is considered as one of the most important pathomechanisms. Astaxanthin (ATX) exhibits strong antioxidant activity in kinds of experimental models. However, the potential of ATX against Hcy-induced neurotoxicity has not been well explored yet. Herein, the neuroprotective effect of ATX against Hcy-induced neurotoxicity in rat hippocampal neurons was examined, and the underlying mechanism was evaluated. The results showed that ATX pre-treatment completely reversed Hcy-induced neurotoxicity through inhibiting cell apoptosis in rat primary hippocampal neurons. The mechanical investigation revealed that ATX effectively blocked Hcy-induced mitochondrial dysfunction by regulating Bcl-2 family and opening of mitochondrial permeability transition pore (MPTP). ATX pre-treatment also attenuated Hcy-induced oxidative damage via inhibiting the release of intracellular reactive oxide species (ROS) and superoxide anion through regulating MPTP opening. Moreover, normalization of MAPKs and PI3K/AKT pathways also contributed to ATX-mediated protective effects. Taken together, these results above suggested that ATX has the potential to reverse Hcy-induced neurotoxicity and apoptosis by inhibiting mitochondrial dysfunction, ROS-mediated oxidative damage and regulation of MAKPs and AKT pathways, which validated the strategy of using ATX could be a highly effective way in combating Hcy-mediated neurological disorders.

## Introduction

Homocysteinemia (Hcy) has been well demonstrated as an independent risk for human neurological diseases, including cerebrovascular diseases, neurodegenerative diseases, central nervous system demyelinating diseases, epilepsy, and peripheral neuropathy^1–5^. Oxidative stress, apoptosis, mitochondrial dysfunction, and interference with N-methyl-D-aspartic acid (NMDA) receptor all contribute to Hcy-mediated pathomechanism for neurological disorders^6^. Reactive oxygen species (ROS)-mediated oxidative damage can damage neurons, induce neural apoptosis or/and necrosis, and inhibition of ROS-mediated oxidative damage has been accepted as an effective strategy in clinic^7,8^. However, Hcy-induced oxidative damage and underlying mechanism remain elusive.

Astaxanthin (ATX) main from *Haematococcus pluvialis* exerts strong antioxidant effects in many cells and animals experimental models^9^. Increasing evidences have confirmed that ATX had the potential to antagonism oxidative damage-mediated disease of cardiovascular system and nervous system^10,11^. Importantly, it is reported that ATX could easily cross the brain-blood barrier (BBB) and show novel neuroprotective effects against neural damage involving anti-oxidation, anti-inflammation, and anti-apoptosis^12–14^. Our previous study revealed that ATX significantly inhibited ROS-mediated oxidative damage and apoptosis in human myocardial cells in vitro and in vivo^15^. However, little information about ATX-mediated neuroprotective effect against Hcy-induced neural toxicity is available, and the underlying mechanism remains to be explored.

## Results

### ATX reverses Hcy-induced neuronal toxicity

**Fig. 1:**
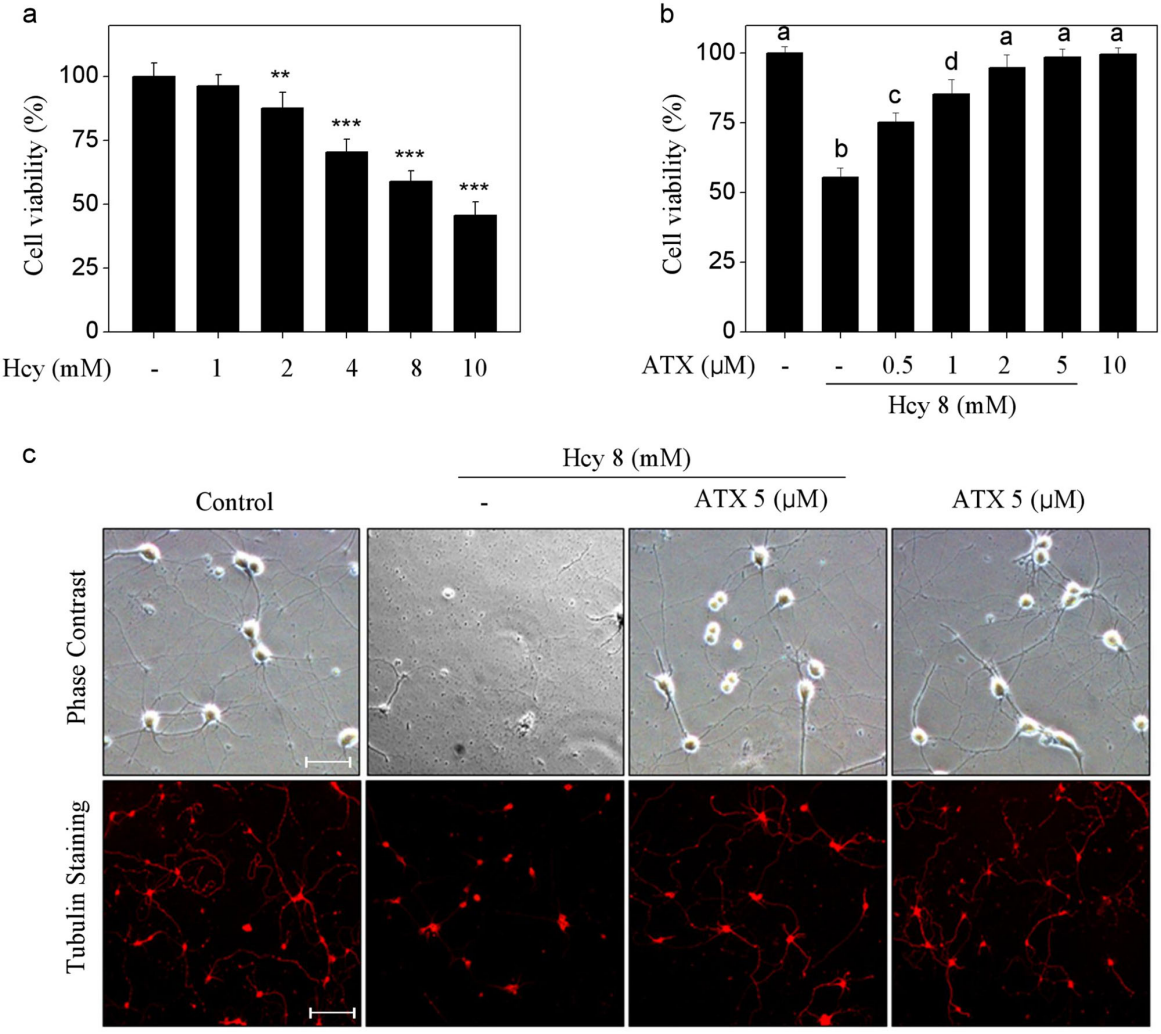
ATX reverses Hcy-induced neuronal toxicity. **a** Neural toxicity of Hcy towards primary neurons. Neurons were treated with 1–10 mM Hcy for 24 h, and neural viability was detected by CCK-8 assay. **b** ATX reversed Hcy-induced neural toxicity. Neurons were pre-treated with 0.5–5 μM ATX before Hcy co-treatment. **c** Morphological changes. Neural morphological changes with or without tubulin staining were observed by phase contrast and fluorescence microscope (magnification, ×200). All data and images were obtained from three random experiments. Scale bar in figures indicates 50 μm. Bars with “*”, “**” and “***” represent the *P* *<* 0.05, *P* *<* 0.01 and *P* *<* 0.001, respectively. Bars with different letters indicate the statistic difference at *P* < 0.05

Primarily, the neuronal toxicity of Hcy towards rat primary hippocampal neurons was screened. As shown in Fig. 1a, Hcy alone significantly inhibited neurons viability with a dose-dependent manner. For example, treatment of neurons with 4, 8, and 10 mM Hcy significantly inhibited cell viability from 100% (control) to 70.3, 58.9, and 45.6%, respectively. However, pre-treatment of neurons with ATX completely reversed Hcy-induced neurons killing. As shown in Fig. 1b, pre-treatment of neurons with 0.5, 1, and 2 μM ATX effectively prolonged the neurons viability from 58.9% (Hcy, 8 mM) to 75.3, 85.4, and 94.8%, respectively. ATX (10 μM) alone showed no cytotoxicity towards neurons. Moreover, neurons morphology was also examined. As shown in Fig. 1c, Hcy-treatment markedly damaged the neural network with decreased neurons number and absent axons. However, ATX pre-treatment dramatically improved morphological changes of neurons. The immunofluorescence staining by tubulin, a neuron marker, further vividly confirmed this protective tendency. Taken together, these results suggested that ATX has the potential to reverse Hcy-induced neuronal toxicity in rat primary hippocampal neurons.

### ATX inhibits Hcy-induced neurons apoptosis

**Fig. 2:**
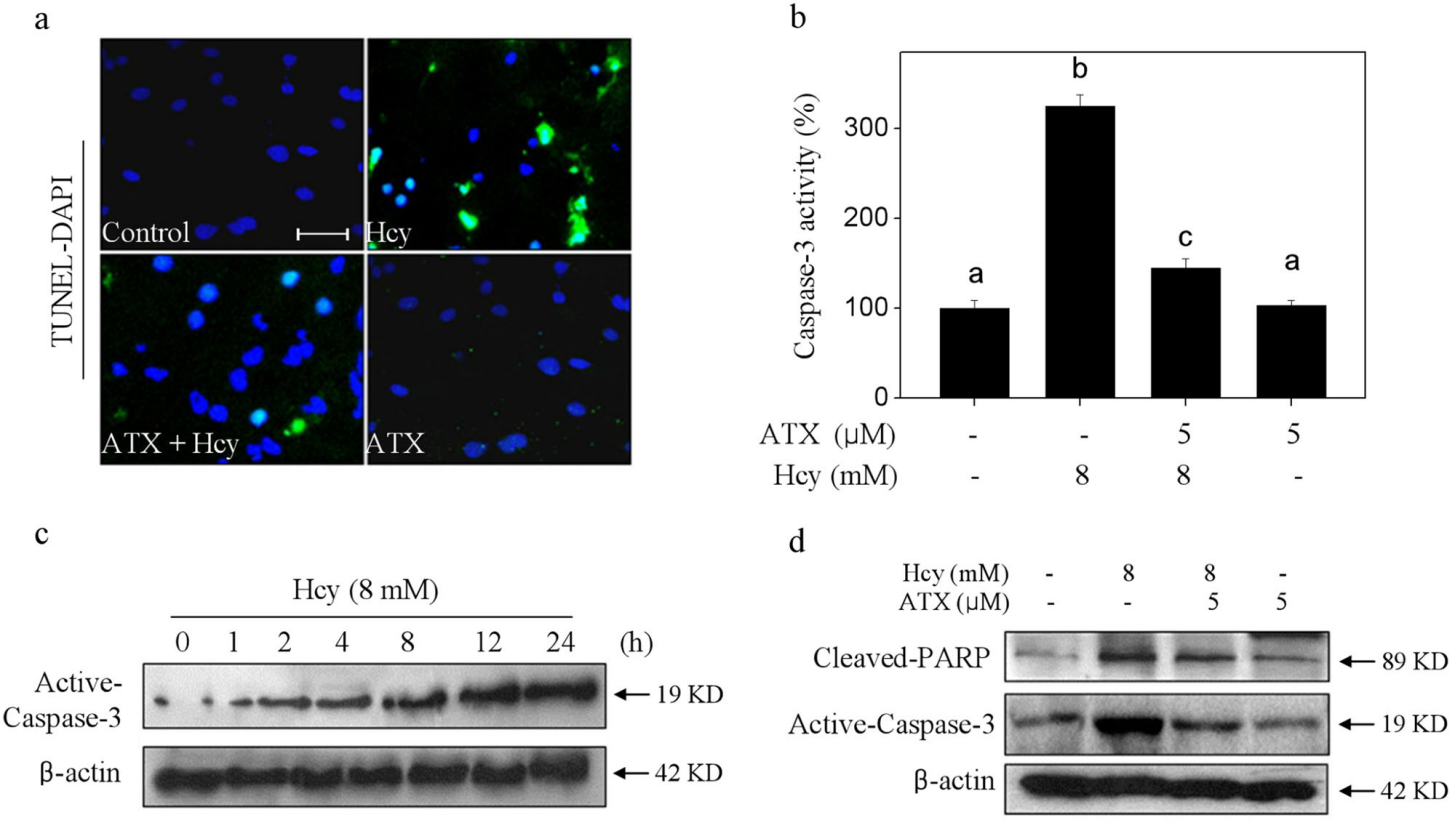
ATX inhibits Hcy-induced neurons apoptosis. **a** Nuclear condensation and DNA fragmentation by TUNEL-DAPI co-staining (magnification, ×200). **b** Caspase-3 activity. **c** Time-course of Hcy on activecaspase-3 expression. **d** ATX attenuated Hcy-induced PARP cleavage and caspase-3 activation. Details of the experiments were conducted according to the section of methods. Scale bar in figures indicates 30 μm. All data and images were obtained from three random experiments. Bars different letters indicate the statistic difference at *P* < 0.05

Hcy-mediated neurons death model was firstly examined by TUNEL-DAPI co-staining. As shown in Fig. 2a, neurons exposed to 8 mM Hcy showed distinct apoptotic characteristics, such as DNA breakage and chromatin condensation, as indicated by the TUNEL-positive neurons. Secondly, Hcy-induced apoptosis was confirmed by caspase-3 activation. As shown in Fig. 2b, Hcy-treatment induced significant caspase-3 activation detected by a specific caspase-3 fluorescence substrate. The time-dependent activation of caspase-3 further convinced Hcy-induced apoptosis. Furthermore, the western blotting showed evident PARP cleavage and caspase-3 activation (Fig. 2d), which further confirmed Hcy-induced apoptosis in protein level. However, ATX pre-treatment significantly attenuated Hcy-induced apoptosis, as demonstrated by the decrease of TUNEL-positive cells, caspase-3 activation and PARP cleavage. Taken together, these results indicated that ATX has the potential to inhibit Hcy-induced neurons apoptosis.

### ATX blocks Hcy-induced mitochondrial dysfunction by regulating Bcl-2 family and MPTP opening

**Fig. 3:**
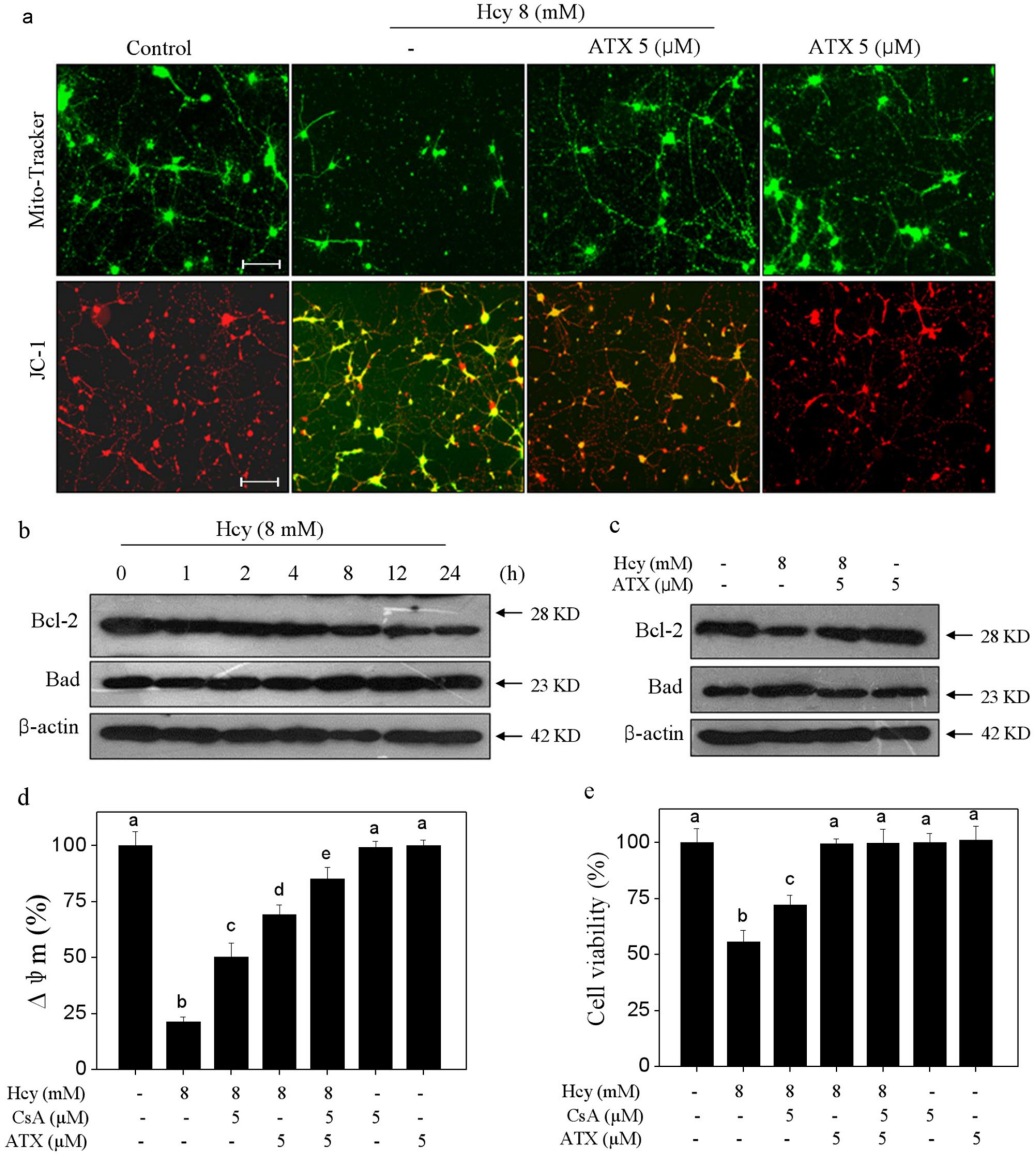
ATX Blocks Hcy-induced Mitochondrial dysfunction by regulating Bcl-2 family and opening of MPTP. **a** ATX blocked Hcy-induced mitochondrial morphological changes and loss of mitochondrial membrane potential (Δψm) (magnification, ×200). **b** Time-course effect of Hcy on Bcl-2 and Bad expression. **c** ATX blocked Bcl-2 family imbalance. **d** Effect of CsA on Δψm in neurons. **e** Effect of CsA on neural viability. Neurons were pre-treated with 5 μM CsA for 2 h before combined treatment. Cell viability and Δψm were detected by CCK-8 assay and JC-1 staining, respectively. Details of experiments were conducted according to the section of methods. Scale bar in figures indicates 50 μm. All data and images were obtained from three random experiments. All images were obtained from three random experiments. Bars different letters indicate the statistic difference at *P* < 0.05

Mitochondria plays key role in regulating intrinsic mitochondria-mediated signaling pathways. Hence, mitochondrial function was evaluated in Hcy-treated neurons. As shown in Fig. 3a, the Mito-Tracker staining (green) showed that Hcy-treatment severely decreased the mitochondria number and fragmented mitochondria-containing filamentous axons. The JC-1 staining showed that Hcy-treatment obviously caused the depletion of mitochondrial membrane potential (Δψm) as indicated by the fluorescent shift from red to green. However, ATX pre-treatment observably blocked Hcy-induced mitochondrial morphological changes and loss of Δψm, respectively. Bcl-2 family, including pro-apoptotic and anti-apoptotic proteins, acts essential role in regulating mitochondrial membrane permeability. Hence, two Bcl-2 family members were examined in Hcy-treated neurons. As shown in Fig. 3b, exposure of cells to 8 mM Hcy significantly downregulated the Bcl-2 expression, but upregulated Bad expression with a time-dependent manner. However, ATX pre-treatment significantly inhibited Bcl-2 family imbalance in Hcy-treated neurons. To further emphasize the role of mitochondria, cyclosporine (CsA), an inhibitor of mitochondrial permeability transition pore (MPTP) was employed. As shown in Fig. 3d, e, MPTP inhibition by CsA significantly improved the ∆ψ_m_ and cell viability in Hcy-treated neurons. Combined treatment of CsA and ATX achieved enhanced improvement of ∆ψ_m_ and neural viability, indicating that ATX can act as a natural inhibitor of MPTP to regulate mitochondria-mediated apoptosis. Taken together, these results clearly suggested that ATX has the potential to block Hcy-induced mitochondrial dysfunction through regulating Bcl-2 family and MPTP opening.

### ATX attenuates Hcy-induced oxidative damage through inhibiting ROS accumulation

**Fig. 4:**
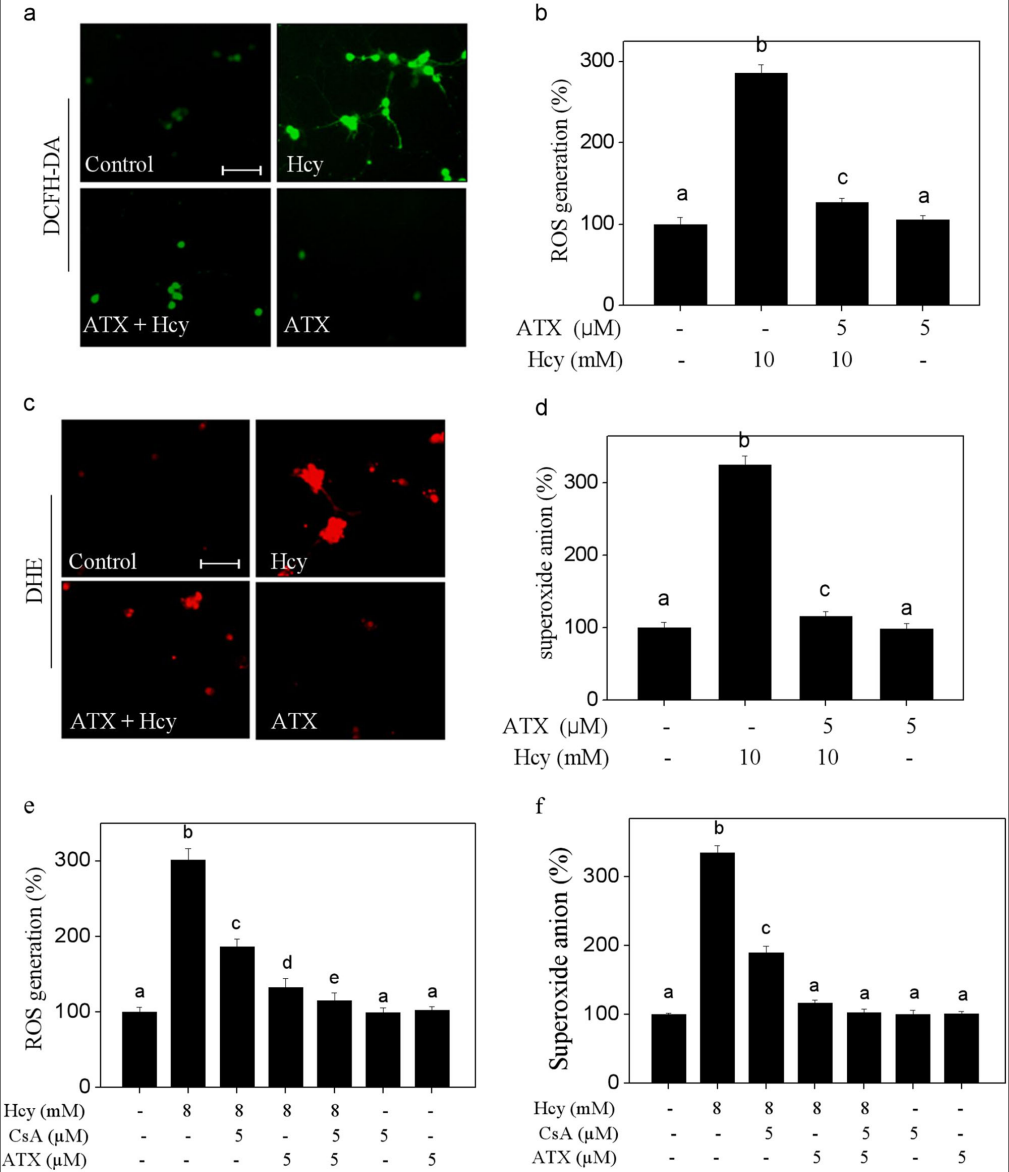
ATX Attenuates Hcy-induced accumulation of ROS and superoxide anion. **a** Detection of ROS. **b** Quantitative analysis of ROS generation. **c** Detection of superoxide anion. **d** Quantitative analysis of superoxide anion generation. ROS and superoxide anion were detected by HCFH-DA and DHE probes (magnification, ×200), respectively as described in the section of methods. **e** Effect of CsA on ROS generation in neurons. **f** Effect of CsA on superoxide anion in neurons. Neurons were pre-treated with 5 μM CsA for 2 h before combined treatment. Generation of ROS and superoxide anion was detected by DCFH-DA and DHE staining, respectively. Scale bar in figures indicates 50 μm. All data and images were obtained from three random experiments. Bars with different letters indicate the statistic difference at *P* < 0.05

**Fig. 5:**
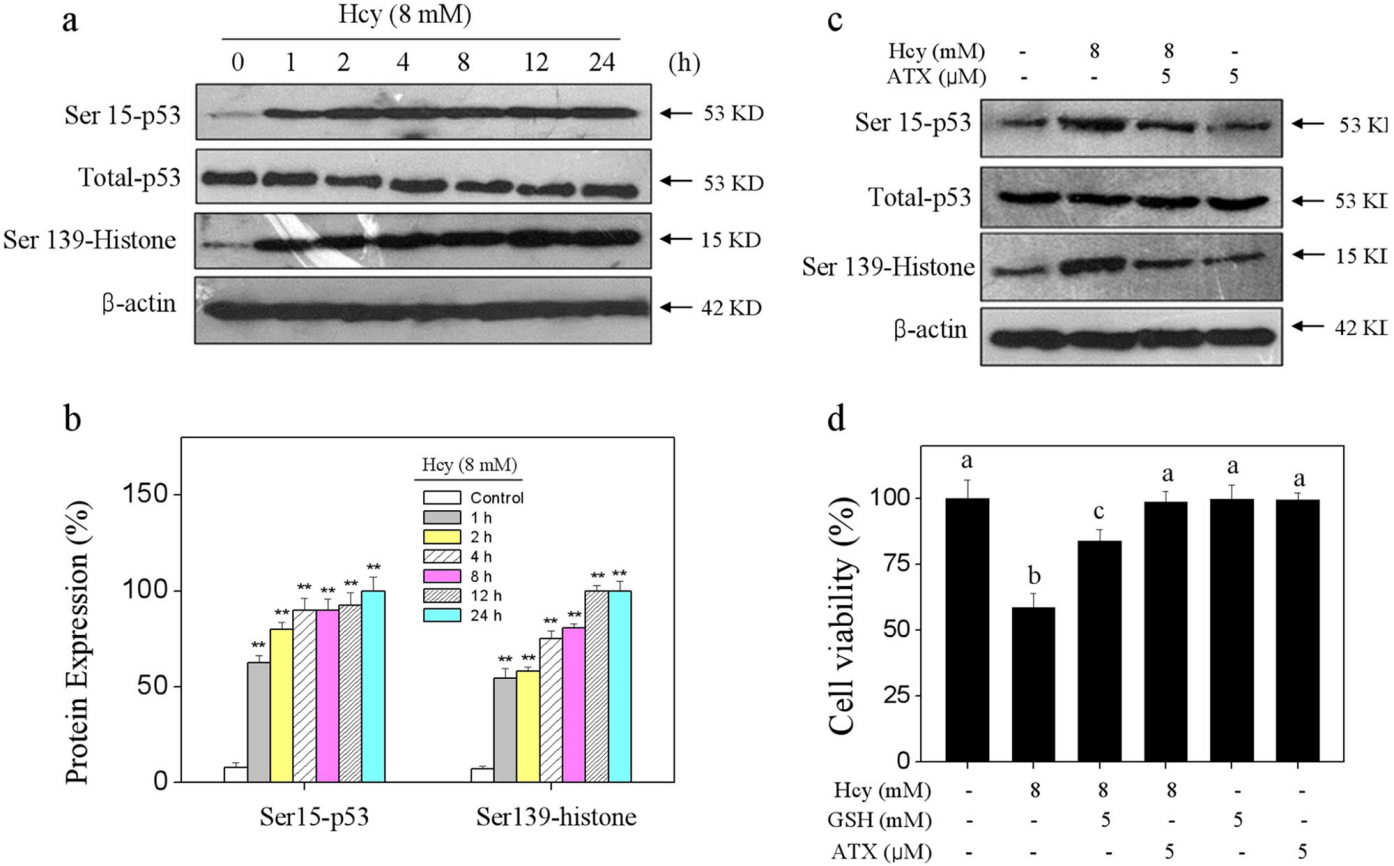
ATX attenuates Hcy-induced oxidative damage. **a** Time-course of Hcy on p53 and histone phosphorylation. **b** Quantitative analysis of Ser15-p53 and Ser139-histone expression. **c** ATX attenuated Hcy-induced p53 and histone phosphorylation. **d** Effect of ATX on Hcy-induced neural toxicity. Neurons were pre-treated with 5 mM GSH for 2 h and co-treated with GSH for 24 h. Neural viability was detected by CCK-8 assay. All data and images were obtained from three random experiments. Bar with “**” or different letters indicate the statistic difference at *P* < 0.05

Induction of oxidative damage is accepted as one of the most important mechanisms of cell death^8^. Hence, the oxidative status was evaluated in Hcy-treated neurons. Initially, the intercellular ROS generation was detected by two specific probes. As shown in Fig. 4, the DCFH-DA and DHE staining showed that Hcy-treatment caused notable ROS and superoxide anion accumulation, as reflected by the enhanced green and red fluorescence, respectively. The quantitative analysis results further confirmed Hcy-induced ROS and superoxide anion overproduction. However, ATX pre-treatment significantly repressed Hcy-induced overproduction of ROS and superoxide anion. To track the ROS source, CsA (a MPTP inhibitor) was employed. As shown in Fig. 4e, f, MPTP inhibition by CsA effectively inhibited the accumulation of ROS and superoxide anion in Hcy-treated neurons. Combined treatment of CsA and ATX achieved enhanced inhibition of ROS and superoxide anion, indicating that ATX can act as a natural MPTP inhibitor to inhibit the release of ROS. Secondly, Hcy-induced oxidative damage was examined by western blotting using specific DNA damage markers. As shown in Fig. 5a, Hcy-treatment time-dependently triggered the phosphorylation of Ser15-p53 and Ser139-H_2_A. The quantitative analysis results further confirmed Hcy-induced DNA damage. ROS inhibition by (glutathione) effectively inhibited Hcy-induced neurons killing, indicating that Hcy-induced neurons cytotoxicity with a ROS-dependent manner. However, ATX pre-treatment dramatically attenuated Hcy-induced DNA damage and neural toxicity. Taken together, these results revealed that ATX had the potential to attenuate Hcy-induced oxidative damage by inhibiting ROS release through regulating MPTP opening.

### Contribution of MAPKs and AKT Pathways

**Fig. 6:**
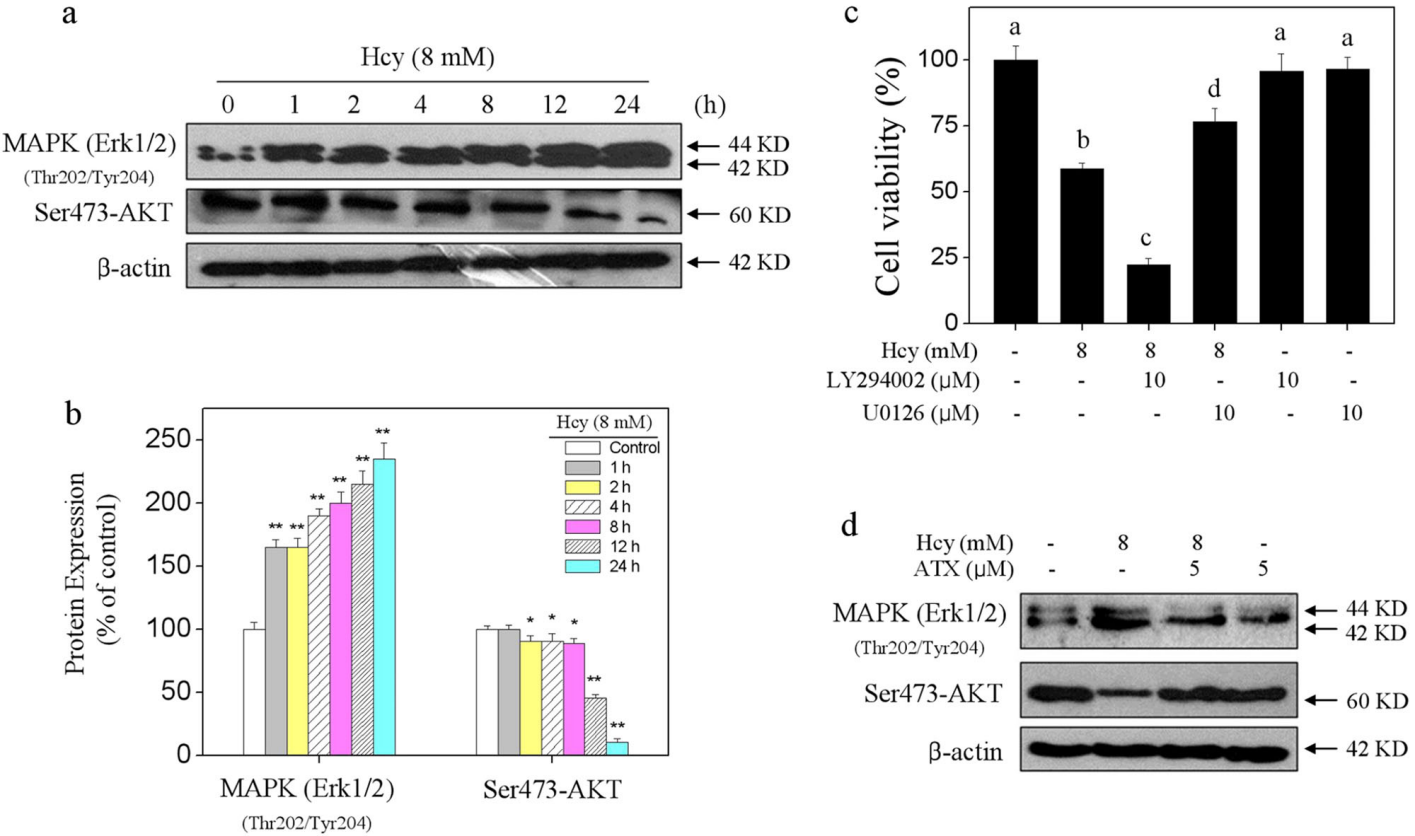
Contribution of MAPKs and AKT pathways. **a** Time-course of Hcy on MAPK (ERK1/2) and AKT phosphorylation. **b** Quantitative analysis of Thr202/Tyr204-MAPK (ERK1/2) and Ser473-AKT expression. **c** Effects of LY294002 (AKT inhibitor) and U0126 (ERK inhibitor) on Hcy-induced neural toxicity. Neurons were pre-treated with 10 μM LY294002 or U0126 for 2 h and co-treated with 8 mM Hcy for 24 h. **d** ATX attenuated Hcy-induced MAPK (ERK1/2) and AKT phosphorylation. All data and images were obtained from three random experiments. Bar with “**” or different letters indicate the statistic difference at *P* < 0.05

MAPKs and PI3K/AKT pathways are of significance in regulating cell growth and cell death. Therefore, ERK and AKT as one of two important members were investigated. As shown in Fig. 6a, Hcy-treatment time-dependently activated ERK at site of Thr202/Tyr204. Ser473-AKT showed continuous inactivation after Hcy treatment. The quantitative analysis results further confirmed this effect of Hcy on the phosphorylation of ERK and AKT (Fig. 6b). To further character the importance of Thr202/Tyr204-ERK and Ser473-AKT, LY294002 (AKT inhibitor) and U0126 (ERK inhibitor) were conducted to investigate their contribution. As shown in Fig. 6c, inhibition of MAPK (ERK1/2) by U0126 significantly suppressed Hcy-induced neurons toxicity. Addition of LY294002 (AKT-upstream inhibitor) significantly enhanced Hcy-induced neurons toxicity. The results indicated that Hcy-induced neurons toxicity with an ERK- and AKT-dependent manner. However, ATX pre-treatment normalized ERK and AKK phosphorylation in Hcy-treated neurons (Fig. 6d). Taken together, these results demonstrated that ATX has the potential to rescue Hcy-induced dysfunction of MAPKs and AKT pathways.

## Discussion

ATX exhibits diverse biological activities and has been studied in various experimental models of neurological diseases^12–16^. Many researchers demonstrated that ATX exerts novel neuroprotective effects on both acute injuries and chronic neurodegeneration, such as ischemic stroke, subarachnoid hemorrhage (SAH), Alzheimer’s disease (AD), and Parkinson’s disease (PD)^12,17,18^. However, ATX-mediated neuroprotection and the underlying mechanism against Hcy-induced neurotoxicity and apoptosis have not been well explored yet.

Primary rat hippocampal neurons as the optimal model to explore the neuroprotective mechanism were commonly used in basic research^19^. Apoptosis, a programmed cell death, plays pivotal role in maintaining the homeostasis^20^. Caspase, a family of cysteine proteases, can trigger apoptotic cascade through the enzymolysis of substrates^21,22^. Caspase-3 activation can promote irreversible PARP cleavage and induce cell apoptosis^23^. In the present study, TUNEL-DAPI co-staining technique showed DNA fragmentation and nuclear condensation in Hcy-treated neuron, which were all typical apoptotic feature. Furthermore, caspase-3 activation and PARP cleavage both confirmed Hcy-induced apoptosis in molecular level. ATX pre-treatment apparently alleviated Hcy-induced caspase-3 activation, PARP cleavage and eventually reversed Hcy-induced neurons apoptosis.

Mitochondrial dysfunction can initiate mitochondria-mediated apoptotic pathway^24^. Large number of studies have proved the relationship of mitochondrial dysfunction and apoptosis in diverse neuronal cellular models, such as rat hippocampal neurons, mouse retinal ganglion cells, mouse embryonic cortical neural progenitor cells, and neuronal PC12 cells^19,25–27^. The opening of mitochondrial permeability transition pore (MPTP) will decrease the mitochondrial membrane potential (Δψm), followed by the release of cytochrome C (cytC), apoptosis-inducing factors (AIFs) and apoptotic protease-activating factor (Apaf)^28–30^. In the present study, ATX pre-treatment significantly reversed Hcy-induced depolarization of Δψm, blocked the mitochondrial morphological changes in Hcy-treated neurons. Bcl-2 family proteins, classified into pro-apoptotic proteins and anti-apoptotic proteins, both affect the Δψm through regulating the MPTP in response to apoptotic stimulation^31^. ATX pre-treatment apparently increased Bcl-xl and Bcl-2 expression, but decreased Bax and Bad expression. The balance of Bcl-2 family after ATX pre-treatment eventually decided the mitochondrial function and the neurons fate^32^. MPTP inhibition by CsA effectively improved the Δψm and the neural viability in Hcy-treated neurons, which further confirmed the significance of mitochondria in Hcy-induced neurotoxicity and apoptosis in neurons. These findings revealed that ATX has the potential to inhibit Hcy-induced mitochondrial dysfunction through regulating Bcl-2 family expression and opening of MPTP.

Oxidative stress induced by Hcy is associated with neuronal system disorders^33,34^. Overproduction of reactive species will lead to the oxidative damage^35,36^. ROS, including hydroxyl radical, singlet oxygen, hydrogen peroxide, and superoxide anion, all play key roles in regulating cellular signaling pathways^37,38^. Overproduced ROS will damage lipids, proteins, and DNA, and finally trigger apoptotic signaling^39^. DNA damage as one of the oxidative damages will activate downstream apoptotic signaling pathways, such as the DNA damaging signal pathway. ATR/ATM, p53, Bcl-2 family/p21 all can be activated in response to oxidative stimuli^40,41^. It is reported that Hcy could induce ROS accumulation and lead to the endothelial dysfunction, and damage the vessel wall^34^. Inhibition of excessive generation of ROS was accepted as one of the most important mechanisms to prevent neurological disease^42^. In the present study, p53 and histone as DNA damage markers were both activated after Hcy treatment^43^. ATX significantly blocked Hcy-induced oxidative damage by eliminating ROS accumulation, indicating that ATX can act as ROS inhibitor to attenuate Hcy-induced oxidative damage. Meanwhile, MPTP inhibition by CsA effectively inhibited the accumulation of ROS and superoxide anion in Hcy-treated neurons, which revealed that mitochondria was the main source of ROS in Hcy-treated neurons, and ATX can act as a potential inhibitor of MPTP to inhibit the ROS release and improve the oxidative status.

MAPKs and PI3K/AKT pathways both play key role in maintenance of homeostasis of the central nervous system^44–48^. ERK and AKT as two important members can regulate apoptosis and cell survival through phosphorylation of a variety of substrates^44–48^. In the present studies, Hcy treatment time-dependently activated ERK phosphorylation, but decreased AKT phosphorylation. Moreover, pre-treatment of ERK and AKT inhibitors effectively inhibited and enhanced Hcy-induced neurons toxicity, respectively. These results suggested that regulation of ERK and AKT contributed to Hcy-induced neurons toxicity. Importantly, pre-treatment with ATX normalized the function of MAPK and AKT pathways, indicating that ERK and AKT both contributed to ATX-mediated neuroprotection against Hcy-induced neural toxicity.

In summary, our findings investigated the in vitro neuroprotective effects and mechanism of ATX against Hcy-induced neural toxicity, and the results suggested that ATX has the potential to reverse Hcy-induced neurotoxicity and apoptosis by inhibiting mitochondrial dysfunction, ROS-mediated oxidative damage and regulation of MAKPs and AKT pathways, which validated the strategy of using ATX could be a highly effective way in combating Hcy-mediated neurological disorders.

## Materials and methods

### Chemicals

MTT, Mitro-tracker probes, JC-1 probes, DCFH-DA probes, and other reagents were purchased from Sigma. TUNEL-DAPI co-staining kit was purchased from Roche. Bicinchoninic acid (BCA) assay kit was purchased from Beyotime Institute of Biotechnology. All antibodies used in this study were purchased from Cell Signaling Technology (Beverly, MA, USA). Dulbecco’s modification of Eagle’s medium (DMEM) and fetal bovine serum (FBS) were purchased from Invitrogen. All solvents used were of high-performance liquid chromatography (HPLC) grade.

### Culture of rat primary hippocampal neurons

Primary hippocampal neurons were dissociated from neonatal (one day) Sprague-Dawley rats. Briefly, neonatal rats were anesthetized with 2% isoflurane and the hippocampus was dissected and digested with 0.2% trypsin for 30 min at 37 ℃. Neurons were seeded in 0.01% poly-l-lysin-coated 6-well plate with B27. Medium was replaced every two days, and after 7 days growth, neuron were treated with Hcy or/and ATX.

### Neural viability assay

Neurons were treated with 1-10 mM Hcy or 0.5-10 μM ATX for 24 h. For combined treatment, neurons were pre-treated with 0.5–5 μM ATX for 2 h and co-treated with 8 mM Hcy for 24 h. Treated neurons were placed into 96-well plate, and neurons viability was detected by CCK-8 assay according to the manufacturer’s instruction. The neuron viability was expressed as % of control. Neuron morphology was examined by phase contrast microscope. Meanwhile, tubulin (a neuron marker) was used to detect the neuron morphology.

### TUNEL-DAPI staining

Neuron apoptosis was detected by TUNEL-DAPI co-staining kit. Briefly, treated neurons were fixed with 4% paraformaldehyde and permeabilized with 0.1% Triton X-100, then incubated with 100 μl/well TUNEL reaction mixture for 1 h and 1 μg/ml DAPI for 15 min at 37 °C, respectively. Then, neurons were washed with PBS for three times and examined under a fluorescence microscope (magnification,×200). TUNEL-positive neurons indicate the apoptotic neurons.

### Caspase-3 activity

Caspase activity was determined by a fluorometric method. Neurons after treatment were harvested by centrifugation, suspended in cell lysis buffer, and incubated on ice for 1 h. After centrifugation at 11,000 × *g* for 15 min, supernatants were collected, normalized for protein concentration, and measured for caspase activity. Briefly, total proteins (100 μg/ well) were placed in 96-well plate, then specific caspase substrates (Ac-DEVD-AMC for caspase-3) were added. After incubation at 37 °C for 2 h in darkness, caspase activity was detected by fluorescence intensity with the excitation and emission wavelengths set at 380 and 440 nm, respectively. The caspase-3 activity was expressed as percentages of control (as 100%).

### Evaluation of mitochondrial function

Mitochondrial membrane potential (Δψm) was evaluated by JC-1 assay. Briefly, treated neurons were incubated with JC-1 dye (5 μg/ml) for 30 min at 37 °C in darkness. Then, neurons were washed with PBS and visualized by fluorescence microscopy (magnification, ×200). The fluorescence shift from red to green represents the loss of Δψm. Alternation of mitochondrial structure was detected by mito-tracker (mitochondria, green) and DAPI (nucleus, blue) co-staining. Cells after treatment were visualized under a fluorescent microscope (magnification, ×200).

### Evaluation of oxidative status

The intracellular ROS level and superoxide anion were examined by DCFH-DA and DHE probes, respectively. Briefly, treated neurons seeded in 96-well plate were incubated with 10 μM DCFH-DA or DHE at 37 °C for 15 min in the dark. After washing twice with PBS, ROS, and superoxide anion generation were detected under a fluorescent microscope (magnification, ×200).

### Western blotting

Neurons after treatment were collected and lysed in RIPA lysis buffer on ice for 1 h. Total protein was extracted and quantified by BCA assay kit strictly complying with the prospectus. Then, total protein (40 μg/lane) after denaturation was loaded and separated in 10% SDS-PAGE (110 V, 75 min). After electrophoresis, the protein was transferred from the gel onto a nitrocellulose membrane and blocked with 5% non-fat milk for 2 h at room temperature. Then, the membrane was incubated with primary antibodies (1:1000) overnight at 4 °C and second antibodies (1:2000) for 1 h at room temperature. Then, the target protein was scanned on X-ray film using an enhanced chemiluminescence system (Kodak). β-actin was used as the reference band.

### Statistical analysis

All experiments were done at least from three independent experiments. SPSS013.0 software was employed for statistical analysis. The significance between two groups was analyzed by two-tailed Student’s test. The difference among three or more groups was analyzed by multiple comparisons. Bars with “*”, “**” and “***” represent the *P* *<* 0.05, *P* *<* 0.01 and *P* *<* 0.001, respectively. Bars with different characters indicates the statistical different at *P* < 0.05 level, which achieves the multiple comparisons among three or more groups.

## References

[1] Yang B (2014). Prevalence of hyperhomocysteinemia in China: a systematic review and meta-analysis. Nutrients.

[2] Rozycka A, Jagodzinski PP, Kozubski W, Lianeri M, Dorszewska J (2013). Homocysteine level and mechanisms of injury in Parkinson’s disease as related to MTHFR, MTR, and MTHFD1 genes polymorphisms and L-Dopa treatment. Curr. Genom..

[3] Kararizou E (2013). Plasma homocysteine levels in patients with multiple sclerosis in the Greek population. J. Chin. Med Assoc..

[4] Belcastro V (2010). Hyperhomocysteinemia in epileptic patients on new antiepileptic drugs. Epilepsia.

[5] Leishear K (2012). Vitamin B12 and homocysteine levels and 6-year change in peripheral nerve function and neurological signs. J. Gerontol. A. Biol. Sci. Med. Sci..

[6] Moustafa AA, Hewedi DH, Eissa AM, Frydecka D, Misiak B (2014). Homocysteine levels in schizophrenia and affective disorders-focus on cognition. Front. Behav. Neurosci..

[7] Chen P (2017). CQ synergistically sensitizes human colorectal cancer cells to SN-38/CPT-11 through lysosomal and mitochondrial apoptotic pathway via p53-ROS cross-talk. Free. Radic. Biol. Med..

[8] Ray PD, Huang BW, Tsuji Y (2012). Reactive oxygen species (ROS) homeostasis and redox regulation in cellular signaling. Cell. Signal..

[9] Regnier P (2015). Astaxanthin from Haematococcus pluvialis Prevents Oxidative Stress on Human Endothelial Cells without Toxicity. Mar. Drugs.

[10] Fassett RG, Coombes JS (2011). Astaxanthin: a potential therapeutic agent in cardiovascular disease. Mar. Drugs.

[11] Augusti PR (2012). Astaxanthin prevents changes in the activities of thioredoxin reductase and paraoxonase in hypercholesterolemic rabbits. J. Clin. Biochem. Nutr..

[12] Shen H (2009). Astaxanthin reduces ischemic brain injury in adult rats. Faseb. J..

[13] Yamagishi R, Aihara M (2014). Neuroprotective effect of astaxanthin against rat retinal ganglion cell death under various stresses that induce apoptosis and necrosis. Mol. Vis..

[14] Stewart JS, Lignell A, Pettersson A, Elfving E, Soni MG (2008). Safety assessment of astaxanthin-rich microalgae biomass: Acute and subchronic toxicity studies in rats. Food Chem. Toxicol..

[15] Fan CD (2017). Astaxanthin attenuates homocysteine-induced cardiotoxicity in vitro and in vivo by inhibiting Mitochondrial dysfunction and oxidative damage. Front Physiol..

[16] Ranga Rao A, Raghunath Reddy RL, Baskaran V, Sarada R, Ravishankar GA (2010). Characterization of microalgal carotenoids by mass spectrometry and their bioavailability and antioxidant properties elucidated in rat model. J. Agric. Food Chem..

[17] Ying CJ (2015). Anti-inflammatory effect of Astaxanthin on the sickness behavior induced by diabetes mellitus. Cell. Mol. Neurobiol..

[18] Liu X, Osawa T (2009). Astaxanthin protects neuronal cells against oxidative damage and is a potent candidate for brain food. Forum Nutr..

[19] Wang J, Bai X, Chen Y, Zhao Y, Liu X (2012). Homocysteine induces apoptosis of rat hippocampal neurons by inhibiting 14-3-3epsilon expression and activating calcineurin. PLoS. One.

[20] Kerr JF, Wyllie AH, Currie AR (1972). Apoptosis: a basic biological phenomenon with wide-ranging implications in tissue kinetics. Br. J. Cancer.

[21] Vandegriff KD (2014). Impact of acellular hemoglobin-based oxygen carriers on brain apoptosis in rats. Transfusion.

[22] Riedl SJ, Shi Y (2004). Molecular mechanisms of caspase regulation during apoptosis. Nat. Rev. Mol. Cell. Biol..

[23] Wu Q (2014). The dual behavior of PCSK9 in the regulation of apoptosis is crucial in Alzheimer’s disease progression (Review). Biomed. Rep..

[24] Green DR, Kroemer G (2004). The pathophysiology of mitochondrial cell death. Science.

[25] Kim SY (2014). Inhibition of cyclophilin D by cyclosporin A promotes retinal ganglion cell survival by preventing mitochondrial alteration in ischemic injury. Cell. Death. Dis..

[26] Hou Y (2014). Permeability transition pore-mediated mitochondrial superoxide flashes mediate an early inhibitory effect of amyloid beta1-42 on neural progenitor cell proliferation. Neurobiol. Aging.

[27] Li DW (2014). Guanosine exerts neuroprotective effects by reversing mitochondrial dysfunction in a cellular model of Parkinson’s disease. Int. J. Mol. Med..

[28] Li J, Yu W, Li XT, Qi SH, Li B (2014). The effects of propofol on mitochondrial dysfunction following focal cerebral ischemia-reperfusion in rats. Neuropharmacology.

[29] Kim J (2012). Rsk-mediated phosphorylation and 14-3-3varepsilon binding of Apaf-1 suppresses cytochrome c-induced apoptosis. Embo. J..

[30] Norberg E, Orrenius S, Zhivotovsky B (2010). Mitochondrial regulation of cell death: processing of apoptosis-inducing factor (AIF). Biochem. Biophys. Res. Commun..

[31] Cory S, Adams JM (2002). The Bcl2 family: regulators of the cellular life-or-death switch. Nat. Rev. Cancer.

[32] Wang Y (2013). The protective effects of selenium on cadmium-induced oxidative stress and apoptosis via mitochondria pathway in mice kidney. Food Chem. Toxicol..

[33] Dayal S (2004). Cerebral vascular dysfunction mediated by superoxide in hyperhomocysteinemic mice. Stroke.

[34] Cloonan L (2015). Metabolic determinants of white matter hyperintensity burden in patients with ischemic stroke. Atherosclerosis.

[35] Shi H, Liu KJ (2007). Cerebral tissue oxygenation and oxidative brain injury during ischemia and reperfusion. Front. Biosci..

[36] Gutteridge JM, Halliwell B (2010). Antioxidants: Molecules, medicines, and myths. Biochem. Biophys. Res. Commun..

[37] Dickinson BC, Chang CJ (2011). Chemistry and biology of reactive oxygen species in signaling or stress responses. Nat. Chem. Biol..

[38] Idelchik M, Begley U, Begley TJ, Melendez JA (2017). Mitochondrial ROS control of cancer. Semin. Cancer Biol..

[39] Chen T, Wong YS (2009). Selenocystine induces reactive oxygen species-mediated apoptosis in human cancer cells. Biomed. Pharmacother..

[40] Palchaudhuri R, Hergenrother PJ (2007). DNA as a target for anticancer compounds: methods to determine the mode of binding and the mechanism of action. Curr. Opin. Biotechnol..

[41] Pellegata NS, Antoniono RJ, Redpath JL, Stanbridge EJ (1996). DNA damage and p53-mediated cell cycle arrest: a reevaluation. Proc. Natl Acad. Sci. USA.

[42] Lee DH, Kim CS, Lee YJ (2011). Astaxanthin protects against MPTP/MPP+ -induced mitochondrial dysfunction and ROS production in vivo and in vitro. Food Chem. Toxicol..

[43] Sancar A, Lindsey-Boltz LA, Unsal-Kacmaz K, Linn S (2004). Molecular mechanisms of mammalian DNA repair and the DNA damage checkpoints. Annu. Rev. Biochem..

[44] Kyriakis JM, Avruch J (2001). Mammalian mitogen-activated protein kinase signal transduction pathways activated by stress and inflammation. Physiol. Rev..

[45] Tsai MC (2015). Factor VII promotes hepatocellular carcinoma progression through ERK-TSC signaling. Cell Death Discov..

[46] Read DE, Gorman AM (2009). Involvement of Akt in neurite outgrowth. Cell Mol. Life Sci..

[47] Boldt S, Weidle UH, Kolch W (2002). The role of MAPK pathways in the action of chemotherapeutic drugs. Carcinogenesis.

[48] Wang, S. et al. Sotetsuflavone suppresses invasion and metastasis in non-small-cell lung cancer A549 cells by reversing EMT via the TNF-α/NF-κB and PI3K/AKT signaling pathway. Cell Death Discov 10.1038/s41420-018-0026-9 (2018) 10.1038/s41420-018-0026-9 PMC5841291 29531823

