# Correction to: Reversal of homocysteine-induced neurotoxicity in rat hippocampal neurons by astaxanthin: evidences for mitochondrial dysfunction and signaling crosstalk

**DOI:** 10.1038/s41420-019-0140-3

**Published:** 2019-03-01

**Authors:** Xian-jun Wang, Wang Chen, Xiao-ting Fu, Jin-kui Ma, Mei-hong Wang, Ya-jun Hou, Da-chen Tian, Xiao-yan Fu, Cun-dong Fan

**Affiliations:** 1 Department of Neurology, People’s Hospital of Linyi Affiliated to Qingdao University, 276000 Linyi, Shandong China; 2 School of Basic Medicine, Taishan Medical University, 271000 Taian, Shandong China; 3 Faculty of Bioresource Sciences, Akita Prefectural University, 241-438 Kaidobata-Nishi, Shimoshinjo-Nakano, 010-0195 Akita-shi, Akita Japan; 4 Department of Neurology, People’s Hospital of Yishui, 276400 Linyi, Shandong China

**Correction to:** **Cell Death Discovery** (2018)

10.1038/s41420-018-0114-x

published online 22 October 2018

The Article contains an error in Fig. 2a. Two images in Fig. 2a (control group and ATX-treated group) were inadvertently duplicated. The control group and ATX-treated group both showed no significant cell apoptosis, the experimenter misunderstood that the same image should be used. (Note: A similar error by the same experimenter was also found in Fig. 2C in another publication “Natural borneol is a novel chemosensitizer that enhances temozolomide-induced anticancer efficiency against human glioma by triggering mitochondrial dysfunction and reactive oxide species-mediated oxidative damage”, 10.2147/OTT.S174498.

**Fig. 2:**
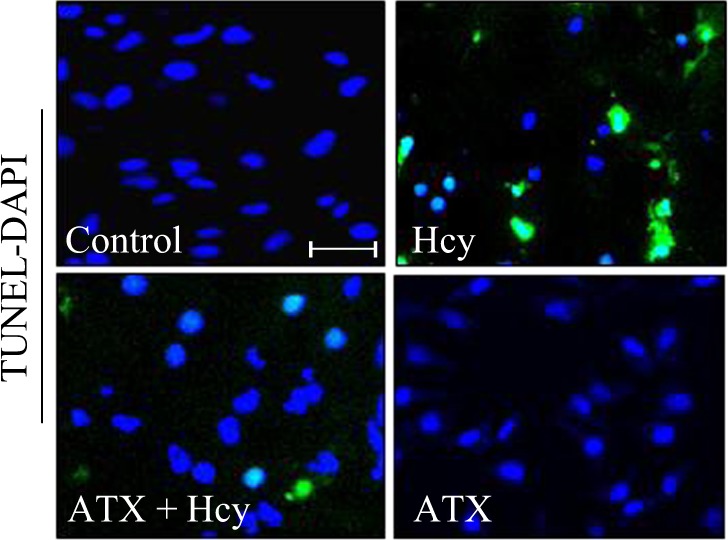
■

The correct Fig. 2a appears below

